# Wildlife Reservoirs of Canine Distemper Virus Resulted in a Major Outbreak in Danish Farmed Mink (*Neovison vison*)

**DOI:** 10.1371/journal.pone.0085598

**Published:** 2014-01-13

**Authors:** Ramona Trebbien, Mariann Chriel, Tina Struve, Charlotte Kristiane Hjulsager, Gitte Larsen, Lars Erik Larsen

**Affiliations:** 1 National Veterinary Institute, Technical University of Denmark, Frederiksberg C, Denmark; 2 Kopenhagen Diagnostics, Glostrup, Denmark; Institut Pasteur, France

## Abstract

A major outbreak of canine distemper virus (CDV) in Danish farmed mink (*Neovison vison*) started in the late summer period of 2012. At the same time, a high number of diseased and dead wildlife species such as foxes, raccoon dogs, and ferrets were observed. To track the origin of the outbreak virus full-length sequencing of the receptor binding surface protein hemagglutinin (H) was performed on 26 CDV's collected from mink and 10 CDV's collected from wildlife species. Subsequent phylogenetic analyses showed that the virus circulating in the mink farms and wildlife were highly identical with an identity at the nucleotide level of 99.45% to 100%. The sequences could be grouped by single nucleotide polymorphisms according to geographical distribution of mink farms and wildlife. The signaling lymphocytic activation molecule (SLAM) receptor binding region in most viruses from both mink and wildlife contained G at position 530 and Y at position 549; however, three mink viruses had an Y549H substitution. The outbreak viruses clustered phylogenetically in the European lineage and were highly identical to wildlife viruses from Germany and Hungary (99.29% – 99.62%). The study furthermore revealed that fleas (*Ceratophyllus sciurorum*) contained CDV and that vertical transmission of CDV occurred in a wild ferret. The study provides evidence that wildlife species, such as foxes, play an important role in the transmission of CDV to farmed mink and that the virus may be maintained in the wild animal reservoir between outbreaks.

## Introduction

Canine distemper virus (CDV) is the etiological agent of one of the most important diseases in wild and domestic predators. The virus infects a broad range of animals belonging to the *Canidae* (dog, fox, wolf etc.) and *Mustelidae* (ferrets, mink, badgers etc.) families [1]–[9]. The virus has a worldwide distribution and can cause disease with high morbidity and mortality in immunologically naïve populations [1], [10].

CDV belongs to the genus *Morbillivirus* within the virus family *Paramyxoviridae.* The virus particle is enveloped and is 150–300 nm in diameter. The virus has a linear, negative-sense, single-stranded, ∼15.7-kb RNA genome encoding the following virus proteins: matrix (M), fusion (F), hemagglutinin (H), nucleocapsid (N), polymerase (L), and phosphoprotein (P) [11]. The H protein is a surface glycoprotein responsible for attachment to the host cell and is an important target for neutralizing antibodies [12]. The H protein is the most variable protein and thus the H gene is the gene most often used to investigate genetic evolution of CDV [2], [13], [14].

Outbreaks of CDV in Danish mink farms with variable severity and prevalence are common (www.vet.dtu.dk). From 2008 to 2010 there were no identified distemper outbreaks and in 2011, CDV was found in only three mink farms between late August and November. A large outbreak of CDV then occurred in farmed mink in the late summer and autumn of 2012 and continued during the first two months of 2013. A total of 64 mink farms were affected in Jutland. In addition, the wildlife population in the same area was affected with a high number of diseased and dead foxes.

The aim of the present study was to track the origin of the virus through a molecular epidemiological examination of the viruses isolated from mink and wildlife species in connection to and preceding the outbreak.

## Materials and Methods

### Samples

Lung tissue samples, which tested positive for CDV by routine diagnostic testing at the National Veterinary Institute (NVI) using specific immunofluorescence histopathology or RT-PCR, were included in the study. A total of 26 mink (*Neovison vison*) samples from 25 different non-vaccinated farms were included. One farm submitted positive samples with 2.5 months interval. Seven CDV positive samples from red wild foxes (*Vulpes vulpes*), two from wild raccoon dogs (*Nyctereutes procyonoides*) and one from wild ferret (*Mustela putorius*) were also included. Sampling dates are indicated in table 1. In addition to the lung samples, fleas (*Ceratophyllus sciurorum*) from a heavily flea infested mink were collected at necropsy as well as fetuses from the wild ferret. Mink samples from CDV outbreaks on mink farms from the years 2004 (n  =  1), 2007 (n  =  3) and 2011 (n  =  1) were also included in the analysis.

**Table 1:** Sample list and accession numbers.

Sampling date	Virus sample	Accession number
25-03-2004	CDV/mink/Denmark/7606338/2004	KF430374
30-07-2007	CDV/mink/Denmark/52–895–1/2007	KF430371
30-07-2007	CDV/mink/Denmark/52–895–4/2007	KF430372
20-09-2007	CDV/mink/Denmark/52–1133–1/2007	KF430373
30-09-2011	CDV/mink/Denmark/52–784/2011	KF430363
29-03-2012	CDV/fox/Denmark/52–1539/2012	KF430362
19-04-2012	CDV/fox/Denmark/52–1541/2012	KF430357
17-06-2012	CDV/fox/Denmark/52–1452/2012	KF430359
20-06-2012	CDV/fox/Denmark/52–1540/2012	KF430361
05-07-2012	CDV/mink/Denmark/52–940–1/2012	KF430348
17-07-2012	CDV/mink/Denmark/52–971–1/2012	KF430353
04-09-2012	CDV/mink/Denmark/52–1077–1/2012	KF430337
05-09-2012	CDV/mink/Denmark/52–1081–1/2012	KF430355
07-09-2012	CDV/mink/Denmark/52–1092–1/2012	KF430351
19-09-2012	CDV/mink/Denmark/52–1122–1/2012	KF430343
25-09-2012	CDV/mink/Denmark/52–1135–1/2012	KF430339
27-09-2012	CDV/mink/Denmark/52–1144–1/2012	KF430356
01-10-2012	CDV/mink/Denmark/52–1148–1/2012	KF430349
02-10-2012	CDV/mink/Denmark/52–1155–1/2012	KF430354
04-10-2012	CDV/mink/Denmark/52–1159–1/2012	KF430352
04-10-2012	CDV/mink/Denmark/52–1164–1/2012	KF430365
05-10-2012	CDV/mink/Denmark/52–1203–1/2012	KF430338
09-10-2012	CDV/mink/Denmark/52–1210–1/2012	KF430347
09-10-2012	CDV/fox/Denmark/52–1288/2012	KF430360
12-10-2012	CDV/mink/Denmark/52–1241–1/2012	KF430367
15-10-2012	CDV/mink/Denmark/52–1240–1/2012	KF430350
18-10-2012	CDV/mink/Denmark/52–1275–1/2012	KF430344
23-10-2012	CDV/mink/Denmark/52–1308–1/2012	KF430345
24-10-2012	CDV/mink/Denmark/52–1318–1/2012	KF430341
25-10-2012	CDV/mink/Denmark/52–1333–1/2012	KF430342
25-10-2012	CDV/mink/Denmark/52–1338–1/2012	KF430340
29-10-2012	CDV/mink/Denmark/52–1356–1/2012	KF430364
01-11-2012	CDV/fox/Denmark/52–1453/2012	KF430358
21-11-2012	CDV/fox/Denmark/52–1585/2012	KF430346
16-01-2013	CDV/mink/Denmark/52–27–1/2013	KF430377
23-01-2013	CDV/mink/Denmark/52–39–1/2013	KF430366
28-01-2013	CDV/mink/Denmark/52–69–1/2013	KF430368
28-01-2013	CDV/raccoon dog/52–79/2013	KF430369
08-02-2013	CDV/mink/Denmark/52–97–1/2013	KF430370
11-04-2013	CDV/racoon dog/52–572–1/2013	KF430376
03-06-2013	CDV/ferret/52–689–1/2013	KF430375

Canine distemper virus samples from mink, foxes, raccoon dogs, and ferret and corresponding accession numbers from Genbank of the hemagglutinin gene.

**Figure 1:**
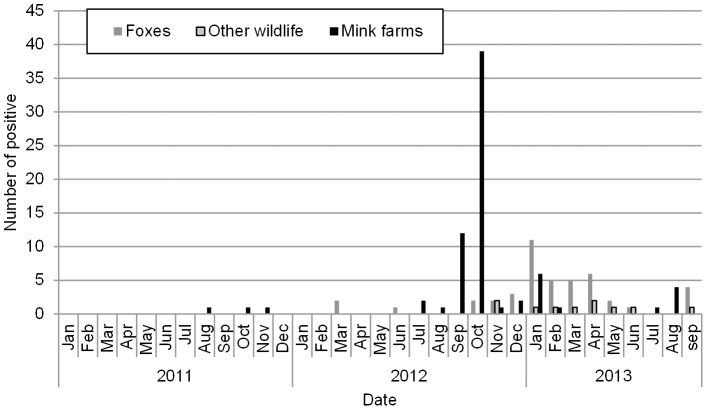
Time course of CDV outbreak in Denmark 2011–2013. The diagram illustrates the outline of the CDV outbreak based on number of positive samples investigated at NVI during 2011–2013 on monthly basis and divided in mink, fox, and other wildlife samples.

Information regarding geographical localization of mink farms and wildlife as well as time of diagnosis was retrieved from the NVI laboratory databases. The time course of the outbreak is illustrated in figure 1.

### Ethics statement

The samples from farmed mink were obtained from dead mink which were submitted to NVI for diagnostic purposes. A submission sheet signed by the farm owner or the farm veterinarian was included in each submission. The submission sheet issued permission to the lab for completing the necessary investigations including test and analysis for CDV. The other source of samples was samples from wildlife species received via the Danish program for surveillance of disease in wildlife species - designated Wildlifehealth.dk. This program is dedicated to research in wildlife disease and passive surveillance of health in the Danish wildlife. The work is done at NVI and is supported by The Danish Nature Agency. The wildlife animals included in the study were found dead and subsequently submitted to the laboratory by employees appointed to collect the animals by the Danish Nature Agency. No endangered species were killed or sacrificed in the frame of the study.

### RNA extraction

Total RNA was extracted from positive lungs by RNeasy Mini Kit (Qiagen, GmbH, Germany) according to the manufacturer’s procedure. 30 mg lung tissue was homogenized with 600 µl RLT-buffer containing β–mercaptoethanol for 3 minutes at 30 hertz on Tissuelyser II (Qiagen, GmbH, Germany). Five fleas from one mink were homogenized in a sterile mortar in 500 µl PBS (0.01 M, pH 7.2); 200 µl of the homogenate were added to 400 µl RLT-buffer containing β–mercaptoethanol. One entire fetus (approximately 40 mg) from the wild ferret was added to 600 µl RLT-buffer containing β–mercaptoethanol and homogenized as described for the lung tissue. A known positive mink lung sample was included as the positive control as well as pure water was used as the negative control. Total RNA was eluted in 60 µl RNase-free water and stored at -80°C until analysis.

### Full-length H-gene RT-PCR and sequencing

To obtain a PCR product for full-length sequencing of the H-gene, a RT-PCR was performed using SuperScript III OneStep RT-PCR System with Platinum Taq High Fidelity (Invitrogen Carlsbad, CA, USA). A total reaction volume of 40 µl was used, which included 2 µl of extracted RNA and 0.6 µM of each primer (Zhao2010fwd and Bolt1997rev, table 2). The amplification temperature profile was 50°C for 30 min for reverse transcription followed by 94°C for 2 min and 40 cycles of 94°C for 30 s, 50°C for 30 s and 68°C for 180 s, and a final extension at 68°C for 10 min. The PCR products were analyzed on 0.8% agarose gels and checked for specific bands of the correct amplicon size (2015 bp). RNase free water was included as the negative control in all RT-PCR runs.

**Table 2:** List of primers used for canine distemper virus H-gene RT-PCR and sequencing.

Primer	Sequence 5′– 3′	Position	Reference
Zhao2010fwd	TTAGGGCTCAGGTAGTCCA	7057–7075	Zhao *et al* 2012 [15]
Bolt1997rev	GGACCTCAGGGTATAGA	9056–9072	Bolt *et al* 1997 [16]
7642fwd	CAGTGGAGCTACTACTTCAGT	7642–7662	This study
7711rev	TGAGATCAAAGACATGGA	7694–7711	This study
8302fwd	GTTGACATTACCTCTAGAT	8302–8320	This study
8380rev	TCCATTCAGTATAACCGGAC	8361–8380	This study

PCR products were purified using High Pure PCR Product Purification Kit (Roche Diagnostics, GmbH, Germany) and cycle sequenced with custom sequencing primers (table 2) at LGC Genomics (GmbH, Germany).

Sequence data analyses were performed using CLC main Workbench 6.6.2 (CLC bio A/S, Aarhus, Denmark). Phylogenetic trees were constructed using a distance-based method with the Neighbor Joining algorithm and bootstrap analysis with 1000 replicates.

### Accession numbers for CDV H-gene sequences

The nucleotide sequences have been submitted to GenBank with accession numbers as indicated in table 1. H-gene sequences of reference viruses from GenBank were used for the phylogenetic analysis and accession numbers are indicated when used.

## Results

### Identity between virus H gene sequences

The nucleotide sequences of the full-length H-gene (1824 bp) of viruses collected during the course of the outbreak in 2012/2013 from the different mink farms were 99.45% to 100% identical when compared pair wisely. Similarly, the virus H-genes of mink, foxes, raccoon dog and wild ferret were 99.45 to 100% identical. The level of nucleotide identity between the Danish outbreak viruses and the vaccine strains Ondersteport and Covac was approximately 91%. Viruses sequenced from the farm that submitted samples twice with 2.5 months interval were 100% identical. When performing a comparison of the outbreak virus sequences to published sequences in Genbank (blast analysis), the viruses showed highest identity to CDV's from wildlife in Germany and Hungary. The highest level of identity (99.29 – 99.62%) was to a virus collected from a red fox in Germany in 2008. These viruses differed with only 7 – 13 nucleotides; however, the nucleotide differences were situated in different regions of the H-gene than seen among the Danish viruses. The Danish mink sample from 2011 also showed a high degree of identity to the 2012/2013 outbreak viruses with 99.62 – 99.95% identity corresponding to 1 – 7 nucleotide differences. Fetuses from the wild ferret contained a virus with a CDV H-gene identical to virus from the lung of the bitch. Interestingly, the CDV H-gene sequence generated from the fleas was 100% identical to the CDV H-gene sequenced from the lung of the flea infested mink.

### Phylogenetic analysis

**Figure 2:**
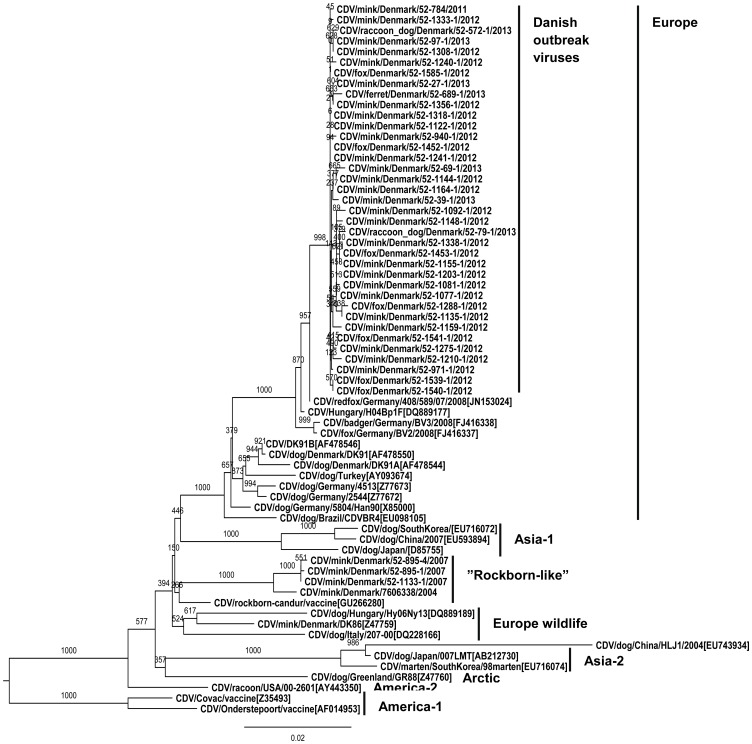
Phylogenetic tree of the CDV H-gene. Phylogenetic tree based on nucleotide sequences of the H-gene (position 1–1824) from outbreak canine distemper viruses and reference viruses. Phylogenetic trees were constructed using distance-based method with the Neighbor Joining algorithm and bootstrap analysis with 1000 replicates.

From the phylogenetic analysis including reference viruses representing the different geographic lineages of CDV, it was clear that the Danish outbreak viruses clustered in the European CDV lineage (figure 2). The viruses were phylogenetically closest related to the above mentioned German and Hungarian viruses. The phylogenetic tree also showed that the viruses from foxes, raccoon dogs and ferret clustered among the Danish mink viruses which strongly suggested that the viruses from mink, foxes, raccoon dogs and ferret had a common ancestor. The Danish CDV virus from 2011 clustered also in the group of the Danish 2012/2013 outbreak viruses. In contrast, the viruses isolated from Danish mink in connection to the outbreaks in 2004 and 2007 clustered separately and together with the Rockborn strain which is the strain in the vaccine Candur® SH+P (Hoechst Roussell Vet GmbH) [17].

### Distribution of mismatches and comparison to epidemiological data

**Figure 3:**
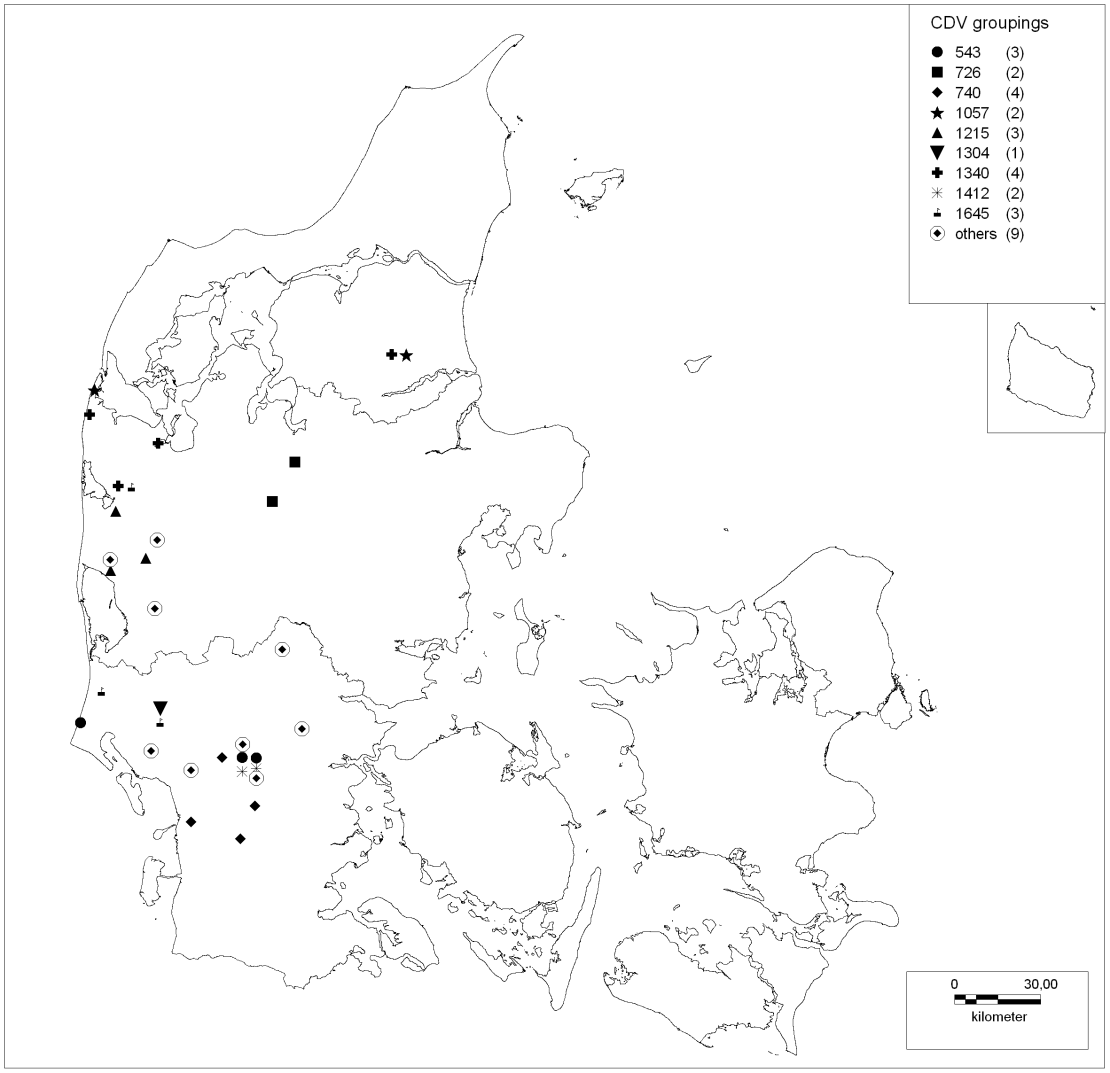
Geographical spread of the nucleotide sequence groupings of the CDV H-gene. Map of Jutland with geographical localization of CDV groupings based on the nucleotide sequence of the H-gene. The symbols represent the following substitutions: circle: C543T; Square: G726T, 1630A, 1814C; Rhomboid: A740C, 1630G, 1814T; Star: T1057C, C1340A, 1630A, 1814C; Upright triangle: T1215C, 1620A, 1814C; Downright triangle: A1304G, 1630A, 1814C; Cross: C1340A, 1630A; Snowflake: C543T, A1412G, 1630G, 1814T; Flag: 1630A, T1645C, 1814C; Open circle with rhomboid: virus sequences not classified into the above mentioned groups.

A detailed analysis of the nucleotide sequence differences of the Danish 2011–13 viruses revealed specific single nucleotide polymorphisms (SNP) (table 3). The Danish 2011–13 virus sequences could be divided mainly into two groups based on the SNP's at positions 1630 (A/G) and 1814 (T/C). Except from one sample, the nucleotide present at these positions seemed to be linked to each other since A in position 1630 always followed C in position 1814 and similarly the G in position 1630 was also linked to T in position 1814. The first samples to be obtained from foxes and mink in September 2011 and March through July 2012, all had the 1630A/1814C combination. The mink sample from 2011 had a sequence identical to the consensus sequence of the outbreak viruses except for one mismatch at position 1620. Some of the virus sequences had similar patterns of single nucleotide polymorphisms (SNP) according to geographical distribution (figure 3). In contrast, there were no SNP's which could group virus according to animal species. The SNP's resulted in amino acid shift at several positions (table 4). Most notable was the motifs in the known signaling lymphocytic activation molecule (SLAM) receptor binding sites 530 and 549. All samples had a G at motif 530, whereas three mink samples had an Y549H substitution.

**Table 3:** Nucleotide differences in the H-gene of outbreak viruses.

	position	210	229	253	313	317	473	543	553	592	678	726	729	737	740	893	920	1057	1104	1124	1152	1153	1156	1215	1278	1296	1304	1340	1353	1377	1412	1440	1455	1462	1608	1620	1630	1643	1645	1657	1751	1814
Virus sample and sampling date	consensus	T	G	C	T	G	G	C	C	G	A	G	C	A	A	A	A	T	T	A	A	A	A	T	A	G	A	C	C	C	A	A	A	G	T	C	G/A	C	T	C	A	T/C
CDV/mink/Denmark/52–784/2011	30-09-2011	.	.	.	.	.	.	.	.	.	.	.	.	.	.	.	.	.	.	.	.	.	.	.	.	.	.	.	.	.	.	.	.	.	.	T	**A**	.	.	.	.	**C**
CDV/fox/Denmark/52–1539/2012	29-03-2012	.	.	.	.	.	.	T	.	.	.	.	.	.	.	.	.	.	.	.	.	.	.	.	.	.	.	.	.	.	.	.	.	.	.	.	**A**	.	.	.	.	**C**
CDV/fox/Denmark/52–1541/2012	19-04-2012	.	.	.	.	.	.	.	.	.	.	.	.	.	.	.	.	.	.	.	.	.	.	.	.	.	G	.	.	.	.	.	.	.	.	.	**A**	.	.	.	.	**C**
CDV/fox/Denmark/52–1452/2012	17-06-2012	.	.	.	.	.	.	.	.	.	.	.	.	.	.	.	.	.	.	.	.	.	.	.	.	.	.	.	.	.	.	.	.	.	.	.	**A**	.	.	.	.	**C**
CDV/fox/Denmark/52–1540/2012	20-06-2012	.	.	.	.	.	.	T	.	.	.	.	.	.	.	.	.	.	.	.	.	.	.	.	.	.	.	.	.	.	.	.	.	.	.	.	**A**	.	.	.	.	**C**
CDV/mink/Denmark/52–940–1/2012	05-07-2012	.	.	.	.	.	.	.	.	.	.	.	.	.	.	.	.	.	.	.	.	.	.	.	G	.	.	.	.	.	.	.	.	.	.	.	**A**	.	.	T	.	**C**
CDV/mink/Denmark/52–971–1/2012	17-07-2012	.	.	.	.	.	.	.	.	.	.	.	.	.	.	.	.	.	.	.	.	.	.	.	.	.	.	.	.	.	.	.	.	.	.	.	**A**	.	C	.	.	**C**
CDV/mink/Denmark/52–1077–1/2012	04-09-2012	.	.	.	.	.	.	.	.	.	.	.	.	.	.	.	.	.	.	.	.	.	.	.	.	.	.	.	.	.	.	.	.	.	.	.	G	.	.	.	.	T
CDV/mink/Denmark/52–1081–1/2012	05-09-2012	.	.	.	.	.	.	.	.	A	.	.	.	.	.	.	.	.	.	.	.	.	.	.	.	.	.	.	.	.	.	.	.	.	.	.	G	.	.	.	.	T
CDV/mink/Denmark/52–1092–1/2012	07-09-2012	.	.	.	.	.	.	.	T	.	.	.	.	.	.	.	.	.	.	C	.	.	.	.	.	.	.	.	.	.	.	.	.	.	.	.	G	T	.	.	.	T
CDV/mink/Denmark/52–1122–1/2012	19-09-2012	.	.	.	.	.	.	.	.	.	.	.	.	.	.	.	.	.	.	.	.	.	.	.	.	.	.	.	.	.	.	.	.	.	.	.	**A**	.	.	.	.	**C**
CDV/mink/Denmark/52–1135–1/2012	25-09-2012	.	.	.	.	.	.	T	.	.	.	.	.	.	.	.	.	.	.	.	.	.	.	.	.	.	.	.	.	.	G	.	.	.	.	.	G	.	.	.	.	T
CDV/mink/Denmark/52–1144–1/2012	27-09-2012	.	.	.	.	.	.	.	.	.	.	.	.	.	.	.	.	C	.	.	.	.	.	.	.	.	.	A	.	.	.	.	.	.	.	.	**A**	.	.	.	.	**C**
CDV/mink/Denmark/52–1148–1/2012	01-10-2012	.	.	.	.	.	.	.	.	.	.	.	.	.	.	T	.	.	.	.	.	C	.	.	.	.	.	.	.	.	.	.	.	.	.	.	G	.	.	.	.	T
CDV/mink/Denmark/52–1155–1/2012	02-10-2012	.	.	.	.	.	.	.	.	.	.	.	.	.	C	.	.	.	.	.	.	.	.	.	.	.	.	.	.	.	.	.	.	.	.	.	G	.	.	.	.	T
CDV/mink/Denmark/52–1159–1/2012	04-10-2012	.	.	.	.	.	.	.	.	.	.	.	.	.	.	.	.	.	C	.	.	.	.	.	.	.	.	A	.	.	.	G	.	.	.	.	**A**	.	.	.	.	T
CDV/mink/Denmark/52–1164–1/2012	04-10-2012	.	.	.	.	.	.	.	.	.	.	.	.	.	.	.	.	.	.	.	.	.	.	.	.	.	.	A	.	.	.	.	.	.	.	.	**A**	.	.	.	.	**C**
CDV/mink/Denmark/52–1203–1/2012	05-10-2012	.	.	.	.	.	.	.	.	.	.	.	T	.	.	.	.	.	.	.	.	.	.	.	.	.	.	.	.	.	.	.	.	.	.	.	G	.	.	.	.	T
CDV/mink/Denmark/52–1210–1/2012	09-10-2012	.	.	T	.	A	.	.	.	.	.	.	.	.	.	.	.	.	.	.	.	.	.	.	.	.	G	.	.	.	.	.	.	.	.	.	**A**	.	C	.	.	**C**
CDV/fox/Denmark/52–1288/2012	09-10-2012	.	.	.	.	.	.	T	.	.	.	.	.	.	.	.	.	.	.	.	.	.	.	.	.	.	.	.	A	.	G	.	.	.	C	.	G	.	.	.	.	T
CDV/mink/Denmark/52–1241–1/2012	12-10-2012	.	.	.	.	.	.	.	.	.	.	.	.	.	.	.	.	.	.	.	.	.	.	.	.	.	.	.	.	.	.	.	.	.	.	.	**A**	.	.	.	.	**C**
CDV/mink/Denmark/52–1240–1/2012	15-10-2012	.	.	.	.	.	.	.	.	.	G	.	.	.	.	.	.	.	.	.	.	.	.	.	.	.	.	.	.	.	.	.	G	.	.	.	**A**	.	.	.	.	**C**
CDV/mink/Denmark/52–1275–1/2012	18-10-2012	.	.	.	.	.	.	.	.	.	.	.	.	.	.	.	.	.	.	.	.	.	.	.	.	.	G	.	.	T	.	.	.	.	.	.	**A**	.	.	.	.	**C**
CDV/mink/Denmark/52–1308–1/2012	23-10-2012	.	.	.	.	.	.	.	.	.	.	.	.	.	.	.	.	.	.	.	.	.	.	C	.	.	.	.	.	.	.	.	.	.	.	.	**A**	.	.	.	.	**C**
CDV/mink/Denmark/52–1318–1/2012	24-10-2012	.	.	.	.	.	.	.	.	.	.	.	.	.	.	.	.	.	.	.	.	.	.	.	.	.	.	.	.	.	.	.	.	.	.	.	**A**	.	.	.	.	**C**
CDV/mink/Denmark/52–1333–1/2012	25-10-2012	.	.	.	.	.	.	.	.	.	.	.	.	.	.	.	.	.	.	.	.	.	.	.	.	T	.	.	.	.	.	.	.	.	.	.	**A**	.	.	.	.	**C**
CDV/mink/Denmark/52–1338–1/2012	25-10-2012	.	.	.	.	.	.	.	.	.	.	.	.	.	C	.	.	.	.	.	G	.	.	.	.	.	.	.	.	.	.	.	.	.	.	.	G	.	.	.	.	T
CDV/mink/Denmark/52–1356–1/2012	29-10-2012	.	.	.	.	.	.	.	.	.	.	T	.	.	.	.	.	.	.	.	.	.	.	.	.	.	.	.	.	.	.	.	.	.	.	.	**A**	.	.	.	.	**C**
CDV/fox/Denmark/52–1453/2012	01-11-2012	.	.	.	.	.	.	.	.	.	.	.	.	.	C	.	.	.	.	.	.	.	.	.	.	.	.	.	.	.	.	.	.	.	.	.	G	.	.	.	.	T
CDV/fox/Denmark/52–1585/2012	21-11-2012	.	.	.	.	.	.	.	.	.	.	.	.	.	.	.	.	.	.	.	.	.	.	.	.	.	.	.	.	.	.	.	.	.	.	.	**A**	.	.	.	.	**C**
CDV/mink/Denmark/52–27–1/2013	16-01-2013	.	.	.	.	.	.	.	.	.	.	T	.	.	.	.	.	.	.	.	.	.	.	.	.	.	.	.	.	.	.	.	.	.	.	.	**A**	.	.	.	.	**C**
CDV/mink/Denmark/52–39–1/2013	23-01-2013	.	.	.	G	.	.	.	.	.	.	.	.	.	.	.	.	.	.	.	.	.	.	.	.	.	.	A	.	.	.	.	.	.	.	.	**A**	.	C	.	.	**C**
CDV/mink/Denmark/52–69–1/2013	28-01-2013	C	.	.	.	.	.	.	.	.	.	.	.	.	.	.	.	C	.	.	.	.	.	.	.	.	.	A	.	.	.	.	.	.	.	.	**A**	.	.	.	G	**C**
CDV/raccoon dog/DK/52–79/2013	28-01-2013	.	A	.	.	.	.	.	.	.	.	.	.	G	C	.	.	.	.	.	.	.	.	.	.	.	.	.	.	.	.	.	.	.	.	.	G	.	.	.	.	T
CDV/mink/Denmark/52–97–1/2013	08-02-2013	.	.	.	.	.	.	.	.	.	.	.	.	.	.	.	.	.	.	.	.	.	.	C	.	.	.	.	.	.	.	.	.	.	.	.	**A**	.	.	.	.	**C**
CDV/racoon dog/DK/52–572–1/2013	11-04-2013	.	.	.	.	.	.	.	.	.	.	.	.	.	.	.	G	.	.	.	.	.	.	C	.	.	.	.	.	.	.	.	.	.	.	.	**A**	.	.	.	.	**C**
CDV/ferret/DK/52–689–1/2013	03-06-2013	.	.	.	.	.	A	.	.	.	.	T	.	.	.	.	.	.	.	.	.	.	T	.	.	.	.	.	.	.	.	.	.	A	.	.	**A**	.	.	.	.	**C**

Positions in the nucleotide sequence of the canine distemper virus H-gene with mismatches to the Danish outbreak virus consensus sequence. The viruses are listed after date of sampling.

**Table 4:** Amino acid differences in the H-protein of the outbreak viruses.

	position	77	85	105	106	158	185	198	246	247	298	307	353	375	385	386	435	447	471	488	530	544	548	549	584	605
Virus sample and sampling date	consensus	D	H	L	R	G	P	V	Q	K	D	D	F	K	K	T	D	S	Q	G	G	T/A	T	Y	D	S/L
CDV/mink/Denmark/52–784/2011	30-09-2011	.	.	.	.	.	.	.	.	.	.	.	.	.	.	.	.	.	.	.	.	T	.	.	.	S
CDV/fox/Denmark/52–1539/2012	29-03-2012	.	.	.	.	.	.	.	.	.	.	.	.	.	.	.	.	.	.	.	.	T	.	.	.	S
CDV/fox/Denmark/52–1541/2012	19-04-2012	.	.	.	.	.	.	.	.	.	.	.	.	.	.	.	G	.	.	.	.	T	.	.	.	S
CDV/fox/Denmark/52–1452/2012	17-06-2012	.	.	.	.	.	.	.	.	.	.	.	.	.	.	.	.	.	.	.	.	T	.	.	.	S
CDV/fox/Denmark/52–1540/2012	20-06-2012	.	.	.	.	.	.	.	.	.	.	.	.	.	.	.	.	.	.	.	.	T	.	.	.	S
CDV/mink/Denmark/52–940–1/2012	05-07-2012	.	.	.	.	.	.	.	.	.	.	.	.	.	.	.	.	.	.	.	.	T	.	.	.	S
CDV/mink/Denmark/52–971–1/2012	17-07-2012	.	.	.	.	.	.	.	.	.	.	.	.	.	.	.	.	.	.	.	.	T	.	H	.	S
CDV/mink/Denmark/52–1077–1/2012	04-09-2012	.	.	.	.	.	.	.	.	.	.	.	.	.	.	.	.	.	.	.	.	A	.	.	.	L
CDV/mink/Denmark/52–1081–1/2012	05-09-2012	.	.	.	.	.	.	I	.	.	.	.	.	.	.	.	.	.	.	.	.	A	.	.	.	L
CDV/mink/Denmark/52–1092–1/2012	07-09-2012	.	.	.	.	.	S	.	.	.	.	.	.	T	.	.	.	.	.	.	.	A	M	.	.	L
CDV/mink/Denmark/52–1122–1/2012	19-09-2012	.	.	.	.	.	.	.	.	.	.	.	.	.	.	.	.	.	.	.	.	T	.	.	.	S
CDV/mink/Denmark/52–1135–1/2012	25-09-2012	.	.	.	.	.	.	.	.	.	.	.	.	.	.	.	.	.	R	.	.	A	.	.	.	L
CDV/mink/Denmark/52–1144–1/2012	27-09-2012	.	.	.	.	.	.	.	.	.	.	.	L	.	.	.	.	Y	.	.	.	T	.	.	.	S
CDV/mink/Denmark/52–1148–1/2012	01-10-2012	.	.	.	.	.	.	.	.	.	V	.	.	.	Q	.	.	.	.	.	.	A	.	.	.	L
CDV/mink/Denmark/52–1155–1/2012	02-10-2012	.	.	.	.	.	.	.	.	T	.	.	.	.	.	.	.	.	.	.	.	A	.	.	.	L
CDV/mink/Denmark/52–1159–1/2012	04-10-2012	.	.	.	.	.	.	.	.	.	.	.	.	.	.	.	.	Y	.	.	.	T	.	.	.	L
CDV/mink/Denmark/52–1164–1/2012	04-10-2012	.	.	.	.	.	.	.	.	.	.	.	.	.	.	.	.	Y	.	.	.	T	.	.	.	S
CDV/mink/Denmark/52–1203–1/2012	05-10-2012	.	.	.	.	.	.	.	.	.	.	.	.	.	.	.	.	.	.	.	.	A	.	.	.	L
CDV/mink/Denmark/52–1210–1/2012	09-10-2012	.	Y	.	Q	.	.	.	.	.	.	.	.	.	.	.	G	.	.	.	.	T	.	H	.	S
CDV/fox/Denmark/52–1288/2012	09-10-2012	.	.	.	.	.	.	.	.	.	.	.	.	.	.	.	.	.	R	.	.	A	.	.	.	L
CDV/mink/Denmark/52–1241–1/2012	12-10-2012	.	.	.	.	.	.	.	.	.	.	.	.	.	.	.	.	.	.	.	.	T	.	.	.	S
CDV/mink/Denmark/52–1240–1/2012	15-10-2012	.	.	.	.	.	.	.	.	.	.	.	.	.	.	.	.	.	.	.	.	T	.	.	.	S
CDV/mink/Denmark/52–1275–1/2012	18-10-2012	.	.	.	.	.	.	.	.	.	.	.	.	.	.	.	G	.	.	.	.	T	.	.	.	S
CDV/mink/Denmark/52–1308–1/2012	23-10-2012	.	.	.	.	.	.	.	.	.	.	.	.	.	.	.	.	.	.	.	.	T	.	.	.	S
CDV/mink/Denmark/52–1318–1/2012	24-10-2012	.	.	.	.	.	.	.	.	.	.	.	.	.	.	.	.	.	.	.	.	T	.	.	.	S
CDV/mink/Denmark/52–1333–1/2012	25-10-2012	.	.	.	.	.	.	.	.	.	.	.	.	.	.	.	.	.	.	.	.	T	.	.	.	S
CDV/mink/Denmark/52–1338–1/2012	25-10-2012	.	.	.	.	.	.	.	.	T	.	.	.	.	.	.	.	.	.	.	.	A	.	.	.	L
CDV/mink/Denmark/52–1356–1/2012	29-10-2012	.	.	.	.	.	.	.	.	.	.	.	.	.	.	.	.	.	.	.	.	T	.	.	.	S
CDV/fox/Denmark/52–1453/2012	01-11-2012	.	.	.	.	.	.	.	.	T	.	.	.	.	.	.	.	.	.	.	.	A	.	.	.	L
CDV/fox/Denmark/52–1585/2012	21-11-2012	.	.	.	.	.	.	.	.	.	.	.	.	.	.	.	.	.	.	.	.	T	.	.	.	S
CDV/mink/Denmark/52–27–1/2013	16-01-2013	.	.	.	.	.	.	.	.	.	.	.	.	.	.	.	.	.	.	.	.	T	.	.	.	S
CDV/mink/Denmark/52–39–1/2013	23-01-2013	.	.	V	.	.	.	.	.	.	.	.	.	.	.	.	.	Y	.	.	.	T	.	H	.	S
CDV/mink/Denmark/52–69–1/2013	28-01-2013	.	.	.	.	.	.	.	.	.	.	.	L	.	.	.	.	Y	.	.	.	T	.	.	G	S
CDV/raccoon dog/Denmark/52–79/2013	28-01-2013	N	.	.	.	.	.	.	R	T	.	.	.	.	.	.	.	.	.	.	.	A	.	.	.	L
CDV/mink/Denmark/52–97–1/2013	08-02-2013	.	.	.	.	.	.	.	.	.	.	.	.	.	.	.	.	.	.	.	.	T	.	.	.	S
CDV/racoon dog/Denmark/52–572–1/2013	11-04-2013	.	.	.	.	.	.	.	.	.	.	G	.	.	.	.	.	.	.	.	.	T	.	.	.	S
CDV/ferret/Denmark/52–689–1/2013	03-06-2013	.	.	.	.	E	.	.	.	.	.	.	.	.	.	S	.	.	.	R	.	T	.	.	.	S

Positions in the amino acid sequence of the canine distemper virus H-gene with mismatches to the Danish outbreak virus consensus sequence. The viruses are listed after sampling date.

## Discussion

Following years with few annual cases of canine distemper virus in Danish farmed mink the virus was absented during the years 2008–2010. In the autumn of 2011, however, CDV was detected in three clinical affected mink farms followed by a large outbreak starting in late summer 2012. This outbreak lasted until the beginning of 2013. Prior to and during the outbreak, farmers and hunters observed diseased and dead foxes in the same geographical areas.

### Molecular epidemiological analysis

In the present study, a molecular epidemiological approach was applied in an attempt to reveal the origin and epidemiology of the severe CDV outbreak by sequencing the gene coding the attachment protein hemagglutinin (H). An intriguing finding was that the H-genes from the different mink farms revealed highly identical sequences, which indicated that the same virus strain was responsible for the outbreaks in the different mink farms; *i.e.* the outbreak was monotypic and probably initiated by one or more introduction(s) of a single virus strain. Furthermore, the H-gene sequences from foxes and raccoon dogs were also inseparable from the mink viruses, confirming that the virus responsible for the wildlife outbreaks was identical to the strain found in mink. Taken together, these results strongly suggested horizontal virus spread among the mink farms and between farmed minks and the wildlife species and vice versa, thereby sustaining an epidemiological link between the wildlife species and farmed mink. It is not possible to determine if a given farm got infected directly from wildlife or by other horizontal routes, but since the movement of animals between farms are minimal it is likely that wildlife species were responsible for most of the outbreaks.

Previous studies have shown the applicability of this molecular epidemiological approach using sequencing of the H-gene to evaluate the evolution of CDV's [18]–[20]. Thus, phylogenetic analyses of the H-gene of global CDV isolates have revealed the presence of eight major geographic genotypes designated as Europe, Europe wildlife, America-1 (vaccine), America-2, Arctic, Asia-1 and Asia-2 [8], [14], [15], [21]. Other minor groups have been characterized e.g. Rockborn like-viruses, African, South American, and Asia-3 (subgroup to Asia-2) [15], [17], [22], [23]. The full–length H-genes of the Danish outbreak viruses from mink, foxes and raccoon dog were all phylogenetically characterized as belonging to the European CDV genotype.

### Species specificity

A range of species belonging to the *Candidae* and *Mustelide* families including, foxes, raccoon dogs, and mink, are susceptible to CDV and crossing of the species barriers are commonly seen. These species jumps have in some cases been linked to substitutions at the amino acid positions 530 and 549 in the SLAM binding region [3], [21]. In the present study, all outbreak sequences from mink, foxes, raccoon dogs and wild ferret had the 530G and 549Y motifs except for three mink samples which had an Y549H substitution. It was, therefore, not possible to distinguish the CDV isolates of the wild canid species (fox and raccoon dog) from the non-canid species (mink) at these positions in the SLAM binding region. This was in accordance with a previous study that showed that CDV sequences from red foxes in Germany and Italy harbored 549H but also the 549Y motif, indicating that both motifs can be found in red foxes [3]. Thus, these signatures could not be used to identify whether the outbreak started in wildlife reservoirs and then spread to mink or if the outbreak started in minks and then spread to wildlife species. Based on the presented and published data it should be reconsidered whether these motifs are true signatures to separate canid and non-canid species.

### Link to previous CDV outbreaks

Archived CDV positive Danish mink samples were sequenced to investigate if the 2012 outbreak could be linked to previous outbreaks. The samples from 2004 and 2007 revealed viruses that had highly identical H-genes, which were clearly different from the 2012 outbreak virus. In contrast, these isolates shared a high level of similarity to the Rockborn strain which was the virus strain included in the MLV CDV vaccine Candur® SH+P (Hoechst Roussell Vet GmbH) [17]. This vaccine strain has previously been shown to be able to revert to virulence, and a study from 2011 revealed that even though the original vaccine was withdrawn from the market in the 1990's, newer vaccines of unknown label contain Rockborn-like viruses [17]. None of these vaccines has been licensed in Denmark; it is, therefore, unclear whether the Danish mink-associated CDV viruses from 2004 and 2007 were wild-type viruses or reverted vaccine viruses. Presently, several commercially available modified-live vaccines are used in mink in Denmark, and the vaccination coverage range from 70–80%.

### Start of the outbreak and origin of the virus

The mink virus collected from the affected farms in 2011 were, however, similar to the 2012 outbreak virus strain with only one nucleotide mismatch to the consensus sequence of the outbreak viruses. This strongly suggested a link between the outbreaks in 2011 and 2012/2013. As the mink samples from 2011 were collected during the autumn, and the outbreak in 2012 started in the mink farms in late summer, there was a gap in time between the outbreaks. Different explanations for the maintenance of the virus strain could be hypothesized e.g. a wildlife reservoir, environmental survival, unnoticed subclinical cases etc. One farm presented CDV infected mink with an interval of 2.5 months with identical viruses; this could indicate environmental survival or subclinical disease at the farm. The most plausible explanation based on sequence data is, however, the hypothesis of a wildlife reservoir. Thus, the first fox to be diagnosed retrospectively was found dead in March 2012 which was four months before the first mink case was diagnosed in July. Foxes from the following months were found positive for CDV, as well. The other wildlife species investigated in the study were observed at a later stage in the outbreak period (nearly a year later) compared to the foxes. The wildlife reservoir explanation is further supported by the finding that the fox and mink viruses were almost identical; furthermore, the motif 1630A/1814C was present in the samples from 2011 and also in positive samples in the first months of 2012. A shift was seen in September 2012 to A1630G/C1814T and subsequently both variants were recognized. These results do not, however, account for the origin of the 2011–13 outbreak virus. A study in Germany has previously shown that CDV is endemic in the red fox population in the federal state Saxony-Anhalt. The study calculated the CDV prevalence to 30.5% based on antigen detection in the red foxes submitted for rabies testing [24]. Blast analysis of the H-gene sequences from the present study indeed revealed that the 2011–13 Danish outbreak virus were 99.62 – 99.95% identical to a virus isolated from a red fox in Germany in 2008. Thus, this virus strain may have circulated unnoticed in a wildlife reservoir in Germany and/or Denmark since 2008 before being transmitted to mink in 2011 *i.e.* by infected wildlife species. It is common to observe wild foxes foraging at mink farms in Denmark after crossing the fence surrounding the farms. However, during the outbreak CDV was also diagnosed in other wild animal species able to cross the fences, such as the raccoon dog and wild ferrets for which reason these species should not be excluded as possible contributing reservoirs.

### Geographical clustering

The tracking of the few nucleotide differences in the analyzed isolates showed SNP's clustering according to geography. This supports the wildlife reservoir theory since animals within the same area will have a closer interaction, and the wildlife species will operate in a limited geographical territory. In contrast, these SNP's analyses failed to show coherence to time of isolation. This could be attributed to the long incubation period of CDV virus and the relative short duration of the outbreak.

### Vertical transmission in ferrets

Fetuses from one wild ferret were CDV positive which shows that CDV can be transmitted vertically from mother to the fetus in this species. Transplacental transmission has previously been documented in dogs [25], [26] but has not previously been reported in wild animals. Vertical transmission of CDV may contribute to the persistence of the virus in wildlife populations.

### CDV in fleas

The identification of identical CDV isolates in a mink submitted for necropsy and fleas collected from the dead mink carcass was intriguing, since CDV has not previously been detected in fleas or in other insects. It is not known if fleas play a significant role for the horizontal transmission of CDV and/or other viruses among mink or between mink and other species such as foxes “visiting” mink farms. Fleas are known to jump from one species to another so this should be subjected to further studies.

## Conclusions

In conclusion, the major outbreak of CDV in Danish farmed mink, in 2012/2013, was caused by a virus strain belonging to the European genotype. The same virus strain was detected in farmed mink and important wildlife reservoir hosts like fox, raccoon dog and wild ferret. The outbreak started most likely in 2011 where identical viruses were obtained from mink. Before the outbreak began in mink farms, in late summer 2012, the virus has most likely been maintained in the wild fox population from which it seemed to have originated. The extensive molecular studies, however, did not reveal if the outbreak started in wildlife reservoirs and then spread to mink or whether it was the opposite scenario. Interestingly, the study revealed that fleas could be a possible vector for CDV. The results of the study add valuable new insight into the epidemiology of this important virus infection and underline the importance of maintaining a high level of biosecurity around mink farms, this in order to avoid introduction of pathogens such as viruses maintained in the wildlife reservoirs.
